# Human iPSC-derived astrocytes transplanted into the mouse brain undergo morphological changes in response to amyloid-β plaques

**DOI:** 10.1186/s13024-021-00487-8

**Published:** 2021-09-25

**Authors:** Pranav Preman, Julia TCW, Sara Calafate, An Snellinx, Maria Alfonso-Triguero, Nikky Corthout, Sebastian Munck, Dietmar Rudolf Thal, Alison M Goate, Bart De Strooper, Amaia M Arranz

**Affiliations:** 1 VIB Center for Brain & Disease Research, Leuven, Belgium; 2 Laboratory for the Research of Neurodegenerative Diseases, Department of Neurosciences, Leuven Brain Institute (LBI), KU Leuven (University of Leuven), Leuven, Belgium; 3 Department of Genetics and Genomic Sciences, Icahn Institute of Genomics and Multiscale Biology, Icahn School of Medicine at Mount Sinai, New York, NY USA; 4 Department of Neuroscience & Friedman Brain Institute, Icahn School of Medicine at Mount Sinai, New York, NY USA; 5 Ronald M. Loeb Center for Alzheimer’s disease, Icahn School of Medicine at Mount Sinai, New York, NY USA; 6 Achucarro Basque Center for Neuroscience, Leioa, Spain; 7 Department of Neurosciences, Universidad del País Vasco (UPV/EHU), Leioa, Spain; 8 VIB Bio Imaging Core, Campus Gasthuisberg, 3000 Leuven, Belgium; 9 Laboratory for Neuropathology, Department of Imaging and Pathology, Leuven Brain Institute (LBI), Department of Pathology, KU Leuven (University of Leuven), University Hospital Leuven, Leuven, Belgium; 10 Dementia Research Institute, University College London, London, UK; 11 Ikerbasque Basque Foundation for Science, Bilbao, Spain

**Keywords:** Human induced pluripotent stem cells (hiPSCs), Astrocytes, Chimeric mouse models, Alzheimer’s disease, Amyloid plaques, Apolipoprotein E (*APOE*)

## Abstract

**Background:**

Increasing evidence for a direct contribution of astrocytes to neuroinflammatory and neurodegenerative processes causing Alzheimer’s disease comes from molecular and functional studies in rodent models. However, these models may not fully recapitulate human disease as human and rodent astrocytes differ considerably in morphology, functionality, and gene expression.

**Results:**

To address these challenges, we established an approach to study human astrocytes within the mouse brain by transplanting human induced pluripotent stem cell (hiPSC)-derived astrocyte progenitors into neonatal brains. Xenografted hiPSC-derived astrocyte progenitors differentiated into astrocytes that integrated functionally within the mouse host brain and matured in a cell-autonomous way retaining human-specific morphologies, unique features, and physiological properties. In Alzheimer´s chimeric brains, transplanted hiPSC-derived astrocytes responded to the presence of amyloid plaques undergoing morphological changes that seemed independent of the *APOE* allelic background.

**Conclusions:**

In sum, we describe here a promising approach that consist of transplanting patient-derived and genetically modified astrocytes into the mouse brain to study human astrocyte pathophysiology in the context of Alzheimer´s disease.

**Supplementary Information:**

The online version contains supplementary material available at 10.1186/s13024-021-00487-8.

## Background

Astrocytes are essential to maintain the homeostasis of the brain, provide trophic support, stimulate synaptogenesis and neurotransmission, and regulate blood-brain-barrier permeability [1, 2]. Impaired astrocytic function contributes to neurological and neurodegenerative disorders including Alzheimer’s disease (AD) [3–8]. In AD patients and AD mouse models, astrocytes undergo robust morphological transformations becoming hypertrophic or atrophic [9–11]. In AD mouse models, astrocytes show an inflammatory and neurotoxic profile together with a reduced expression of genes involved in neuronal support and communication [12–14], and display aberrant calcium dynamics [15]. Moreover, reactive astrocytes in AD mice increase oxidative stress and formation of reactive-oxygen species (ROS), show mitochondrial dysfunction [16] and enhance the release of neurotransmitters including glutamate, GABA and ATP [17]. These morphological, molecular and functional alterations highlight the potential importance of these cells in the pathogenesis and progression of AD.

While transgenic models have provided invaluable tools to study the contribution of astrocytes to AD [13, 18–20], they might insufficiently mimic the human disease, as there are major differences between rodent and human astrocytes. Morphologically, human astrocytes are larger and more complex, having around 10 times more processes than their rodent counterparts [21]. Molecularly, human and mouse astrocytes display different, although overlapping, gene expression profiles [22]. Functionally, human astrocytes propagate calcium waves four-fold faster than rodent ones [21–23], and human and mouse astrocytes show very different responses when exposed to inflammatory stimuli [24, 25].

The ability to generate human induced pluripotent stem cells (hiPSCs) and differentiate them into astrocytes and other CNS cell types provides exciting opportunities to examine AD associated phenotypes *in vitro* and unravel the contribution of astrocytes to AD [26–29]. Yet, hiPSC-derived astrocytes grown in culture lack essential components present in the brain which can induce altered phenotypes and gene expression signatures significantly different from that of primary resting astrocytes in the brain [22, 30]. Therefore, it has proved challenging to advance understanding of human astrocytic function in AD.

To address these challenges, we aimed at developing a chimeric model that allowed studying hiPSC-derived astrocytes in an *in vivo* AD context. We and others have generated chimeric models to study AD by transplanting hPSC-derived neurons or microglia into the brains of suitable AD mice and WT littermates [31–33]. These models have revealed that human neurons and microglia transplanted into the mouse brain respond to pathology differently than their murine counterparts, showing specific vulnerability and transcriptional signatures when exposed to amyloid-β (Aβ) [31, 32]. Moreover, human glia chimeric mice have been generated by Goldman and collaborators to investigate the function of the engrafted cells, mainly NG2 cells (oligodendrocyte progenitors) with variable proportions of oligodendrocytes and astrocytes, in disease relevant conditions such as Huntington disease, Schizophrenia or hypomyelination [34–36]. Yet, to date no studies have analyzed the phenotypes and functional responses of xenografted human astrocytes exposed to Aβ and AD-associated pathology *in vivo*.

We establish here a chimeric model to investigate survival, integration, properties, and responses to Aβ species of human astrocytes expressing *APOE* ε3 (E3) or *APOE* ε4 (E4) variants. We document the engraftment of astrocytes that integrate in a functional way in the mouse host brain and display human-specific morphologies and properties. Transplanted human astrocytes in chimeric AD brains undergo morphological transformations becoming hypertrophic or atrophic, similar to astrocytes in AD patients’ brains [9, 11, 37]. Our results validate the use of chimeric mice as a potential powerful system for studying human astrocyte contribution to AD. We also discuss the variable degree of chimerism from different individual hiPSC lines and limited recovery of transplanted human astrocytes for further molecular analyses which poses a hurdle to fully capture the potential of this approach.

## Results

### Human iPSC-Derived Astrocyte Progenitors Engraft the Mouse Brain and Differentiate into Astrocytes

**Fig. 1:**
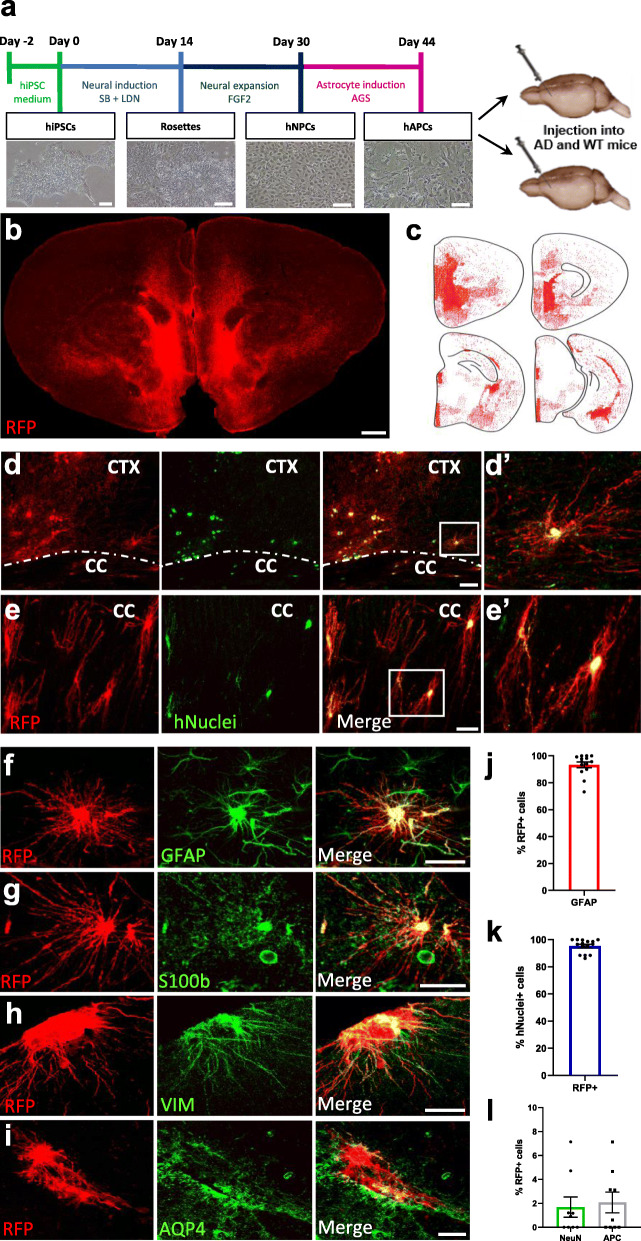
hiPSC-astrocyte progenitors engraft the mouse brain and differentiate into astrocytes within chimeric AD and WT mice.** (a)** Schematics of the differentiation and transplantation procedures. hiPSCs: human induced pluripotent stem cells, hNPCs: human neural progenitor cells, hAPCs: human astrocyte progenitor cells, SB: SB431542, LDN: LDN193189, FGF2: fibroblast growth factor 2, AGS: astrocyte growth supplement. Scale bars: 100 µm. **(b)** RFP staining (red) shows the distribution of hiPSC-derived astrocytes on a coronal brain section of a chimeric mouse five months after transplantation. Scale bar: 200 µm. **(c)** Dot map displaying the widespread distribution of the hiPSC-derived astrocytes (RFP, red) in four coronal sections of this mouse brain. **(d-e)** RFP (red) and hNuclei (green) expressing hiPSC-astrocytes depict a complex fine structure in the cortex (CTX) and corpus callosum (CC) of chimeric mice. Scale bars: 50 µm (**d**), 25 µm (**e**). **(d’-e’)** Enlarged images of the inserts in **d** and **e**. **(f-i)** Engrafted hiPSC-astrocytes (RFP+, red) express GFAP (**f**), S100b (**g**), Vimentin (**h**) and AQP4 (**i**) (green) five months after transplantation. Scale bars: 25 μm. **(j)** Percentage of RFP+ cells expressing GFAP (*n* = 14 mice). **(k)** Percentage of hNuclei+ cells expressing RFP (*n* = 15 mice). **(l)** Percentage of RFP+ cells expressing NeuN and APC (*n* = 9 mice). Data are represented as mean ± SEM

To generate human-mouse astrocyte chimeras, we differentiated human iPSCs (hiPSCs) into astrocyte progenitor cells (hAPCs) *in vitro* [38] (Fig. 1a). After 44 days in culture, td-Tomato expressing hAPCs, which expressed several astrocyte markers (Fig. S1b), were xenografted into the brains of newborn mice (Fig. 1a). We used transgenic Tg (Thy1-APPSw,Thy1-PSEN1*L166P) 21Jckr, also called *APP/PS1*-21 mice [39] crossed with immunodeficient NOD.CB17-*Prkdc*^scid^/J, further called NOD-SCID mice [40], to generate AD mice or wild-type (WT) littermates suitable for grafting experiments [31]. We transplanted hiPSC lines from AD patients carrying the *APOE* E4/E4 alleles and the corresponding corrected *APOE* E3/E3 isogenic lines (Table 1).

**Table 1:** Information on the hiPSC lines.

hiPSC line	hiPSC name	Ethnicity	Gender	Age of onset	Age at skin biopsy	Disease status (CDR at biopsy)	*APOE* genotype	Genetic modification
1	TCW1E33-1F1	Caucasian	F	64	72	AD (2)	E4/E4	E3/E3
2	TCW1E44-2C2	Caucasian	F	64	72	AD (2)	E4/E4	E4/E4
3	TCW2E33-3D11	Caucasian	M	77	80	AD (0.5)	E4/E4	E3/E3
4	TCW2E44-4B12	Caucasian	M	77	80	AD (0.5)	E4/E4	E4/E4
5	TCW2E33-2E3	Caucasian	M	77	80	AD (0.5)	E4/E4	E3/E3
6	TCW2E44-4B1	Caucasian	M	77	80	AD (0.5)	E4/E4	E4/E4
7	TCW3E33-H-2	Caucasian	M	80	83	AD (0.5)	E4/E4	E3/E3
8	TCW3E44-F-2	Caucasian	M	80	83	AD (0.5)	E4/E4	E4/E4

The table shows hiPSC name, patient ethnicity, gender, age of AD onset, age at skin biopsy, disease status (CDR at biopsy), original *APOE* genotype and genetic modification. F female, M male, AD Alzheimer’s disease, *APOE* apolipoprotein, CDR clinical dementia rating, hiPSC human induced pluripotent stem cells. These cells were previously generated and characterized by [29]

Five months after transplantation, immunofluorescence (IF) analysis revealed engraftment of human cells throughout the forebrain (Fig. 1b, Fig. S1c). Human cells were identified based on the expression of the td-Tomato marker RFP and of the human nuclear antigen hNuclei. RFP+ cells infiltrated the cortex, corpus callosum and subcortical areas such as the hippocampus, striatum, thalamus or hypothalamus (Fig. 1c-e). Assessment of the engraftment capacity revealed considerable variation across cell lines (Fig. S1d): we show here examples of robust engraftment, with RFP+ cells both in clusters as well as integrated individually within the mouse brain (Fig. 1b, c), but these results were variable with often lower engraftment capacity at 5 months after transplantation (Fig. S1c, d). Variation was independent of the *APOE* genetic background or the patient (overview in Fig. S1d).

Further analyses revealed that at this stage, human RFP+ cells strongly expressed the astrocyte markers GFAP, S100b, Vimentin and Aquaporin-4 (Fig. 1f-i), the latter largely concentrated at the astrocytic end-feet along the blood vessels (Fig. 1i). Staining with human specific GFAP antibody (hGFAP) confirmed the human origin of the cells (Fig. S3a). Quantification showed that 93 % of the RFP+ hiPSC-cells expressed the astrocyte marker GFAP (Fig. 1j) and 95 % of the hNuclei+ hiPSC-cells co-expressed RFP (Fig. 1k). Thus, the RFP marker was not downregulated, and most of the transplanted cells indeed differentiated into human astrocytes. This was further confirmed as no or only minimal expression (less than 3 %) of neuronal or oligodendroglial markers was observed in RFP+ cells (Fig. 1L, Fig. S3b, c). No differences in cell identity were observed between *APOE* E4/E4 and *APOE* E3/E3 lines or between cells transplanted in WT or AD chimeric mice (Fig. S3d-f). A subset of RFP+ cells identified by their distinct radial glia-like morphology and not expressing GFAP (Fig. S3g-i) often coexisted with RFP+ cells with more complex structures and expressing GFAP and main astrocyte markers (Fig. S3j). These cells were likely in a progenitor state which was also described previously [23, 41].

### Transplanted hiPSC-Derived Astrocytes Integrate Functionally Within the Mouse Brain

**Fig. 2:**
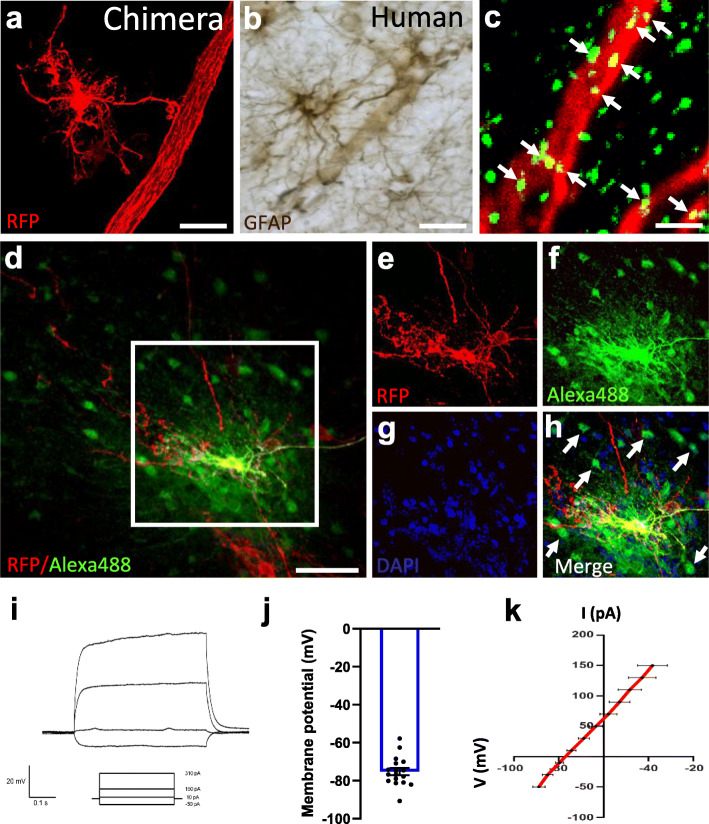
hiPSC-astrocytes integrate functionally within the mouse brain.** (a-b)** A xenografted hiPSC-astrocyte in the chimeric mouse brain (**a**, red) and a GFAP+ cortical astrocyte in the human brain (**b**, brown) contacting blood vessels with their end-feet. Scale bars: 25 μm. **(c)** hiPSC-astrocyte processes (RFP, red) express the gap junction marker Cx43 (green, arrows). Scale bar: 2 μm. **(d)** The gap-junction dye Alexa488 loaded on a hiPSC-astrocyte (RFP+, red) diffuses into RFP- neighboring host cells. Scale bar: 25 μm. **(e-h)** Enlarged views of the area selected in **d**. (**e**) RFP+ hiPSC-astrocyte, (**f**) Alexa488 dye, (**g**) Nuclei stained with DAPI, (**h**) Overlay. Arrows point to Alexa488+ RFP- host cells. **(i-k)** Representative traces of current injection steps of 20mV (**i**), resting membrane potentials (**j**) and current-voltage (I/V) curves (**k**) of hiPSC-astrocytes in the host brain (n = 17 cells from 6 WT mice). Data are represented as mean ± SEM

We assessed morphological and electrophysiological features of individual hiPSC-derived astrocytes in the chimeric brain. We observed hiPSC-astrocytes extending processes that terminated in end-feet contacting mouse host vasculature in the chimeric brain (Fig. 2a) similar to human astrocytes in the human brain (Fig. 2b). Moreover, hiPSC-astrocytes strongly expressed the gap-junction marker Connexin-43 in their processes (Fig. 2c). The gap junctions were functioning, as the Alexa488 dye loaded through the patch clamp pipette on RFP+ astrocytes diffused into neighboring mouse host cells (Fig. 2d-h). Electrophysiological analyses on acute brain slices of chimeric mice at 4–5 months showed that transplanted RFP+ astrocytes displayed properties resembling human astrocytes [42]. Specifically, they displayed non-excitable responses to stimulations with current injection in current clamp mode (Fig. 2i), and human-like resting membrane potentials (Fig. 2j) and linear current to voltage (I/V) curves (Fig. 2k). Human iPSC-astrocytes did not replace the endogenous murine astrocytes and both cell types were found in the chimeric mouse brains (Fig. S3j). These data revealed that the transplanted hiPSC-astrocytes were able to integrate functionally within the mouse host brain, showed human-like physiological features and co-existed with endogenous mouse counterparts.

### Human iPSC-Derived Astrocytes Acquire Human-Specific Morphologies and Features ***In Vivo***

**Fig. 3:**
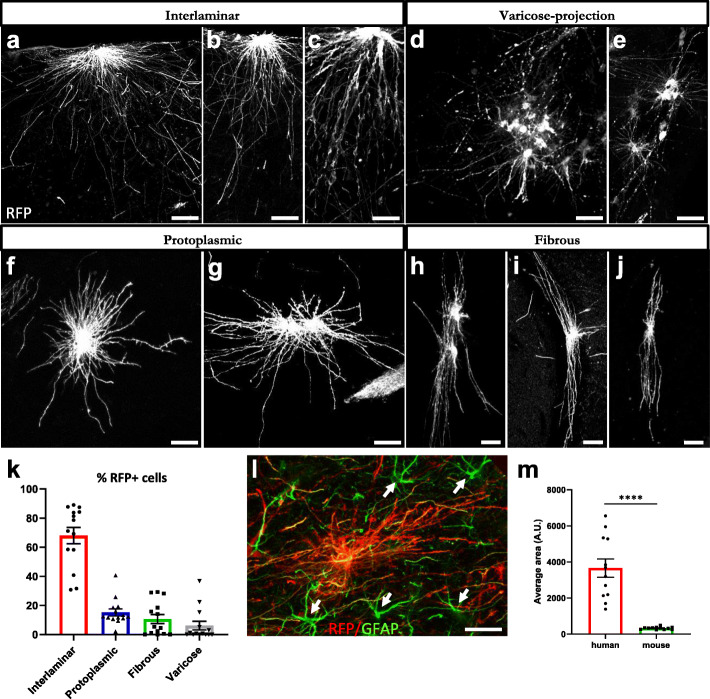
hiPSC-astrocytes recapitulate human morphological subtypes and retain human specific features within the mouse brain.** (a-j)** Representative images of RFP+ (white) interlaminar (**a**-**c**), varicose-projection (**d**-**e**), protoplasmic (**f**-**g**) and fibrous astrocytes (**h**-**j**) in the brain of WT mice five months after transplantation. Scale bars: 25 μm. **(k)** Histogram showing the percentage of RFP+ cells of each astrocyte subtype on the mouse brain (n = 14 mice). Data are represented as mean ± SEM. **(l)** Representative image showing mouse (green, arrows) and hiPSC-astrocytes (red) on a chimeric mouse brain five months after transplantation. Scale bar: 25 μm. **(m)** Histogram plotting the size of hiPSC-derived astrocytes vs. mouse astrocytes on the host brain (*n* = 12 mice). Data are represented as mean ± SEM, Student’s t test: ****p < 0.0001

**Fig. 4:**
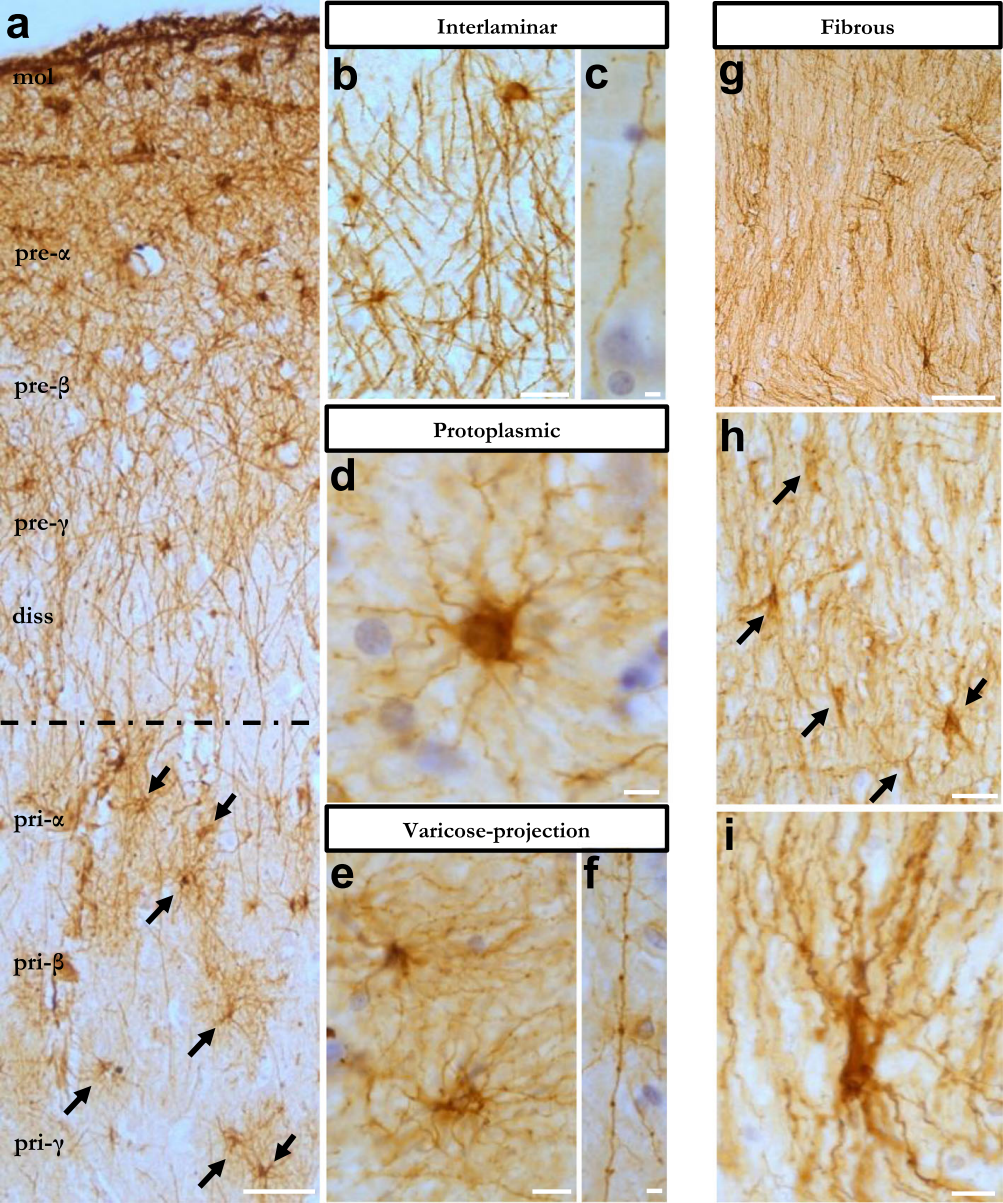
Four subtypes of morphologically defined GFAP+ astrocytes in the human entorhinal cortex and white matter.** (a)** Overview of human entorhinal cortex layers stained with GFAP (brown) to detect astrocytes. Layers molecular to lamina dissecans are mainly composed of subpial interlaminar astrocytes, while layers pri-α to pri-γ are rich in protoplasmic astrocytes (arrows). **(b-f)** Representative images of subpial interlaminar astrocytes (**b**) and their tortuous processes (**c**), protoplasmic astrocytes (**d**), varicose-projection astrocytes (**e**) and their beaded processes (**f**). **(g-i)** Human white matter GFAP+ fibrous astrocytes (**h**-**i**). mol: molecular layer, diss: lamina dissecans. Scale bars: 50 μm in (**a**) and (**g**); 25 μm in (**b**) and (**h**); 10 μm in (**c**-**f**) and (**i**)

We examined the morphological phenotypes of the transplanted astrocytes. Five months after transplantation, four main morphological subtypes of hiPSC-derived astrocytes were identified in the chimeric brains of control WT animals. RFP+ interlaminar astrocytes were frequently observed in superficial layers of the cortex and close to the ventricles, with their small and round cell bodies near the pial surface and their long, unbranched and sometimes tortuous processes descending into deeper layers (Fig. 3a-c). Varicose-projection astrocytes were relatively sparse but easily identified by their bushy appearance and the presence of long processes with regularly spaced beads or varicosities (Fig. 3d, e). Protoplasmic astrocytes were found in deeper layers of the brain and showed the characteristic star-shaped morphology and shorter processes extending in all directions and often contacting the vasculature (Fig. 3f, g). Fibrous astrocytes were found in white matter tracts and presented the typical morphology with small soma and fine, straight and radially oriented processes (Fig. 3h-j). Interlaminar astrocytes were the most abundant subtype of hiPSC-astrocytes in the mouse brain, summing up to 68 % of the RFP+ cells, followed by protoplasmic and fibrous astrocytes (15 and 11 % of the RFP+ cells, respectively). The varicose-projection astrocytes were the less frequent subtype, constituting 6 % of RFP+ cells found in the host brain (Fig. 3k). We confirmed in the brain of human healthy individuals the presence of the same astrocyte subtypes in the entorhinal cortex and in white matter tracts (Table 2, subjects 10–12), when staining with the astrocyte marker GFAP. We found subpial interlaminar astrocytes with their soma in superficial layers of the cortex (molecular layer to pre-α) and long processes extending into deeper layers (Fig. 4a-c), protoplasmic (Fig. 4a, d) and varicose-projection astrocytes (Fig. 4e-f) in deeper layers of the cortex (pri-α to pri-γ) and fibrous astrocytes in white matter tracts (Fig. 4g-i). Of note, hiPSC-astrocytes covered about 15-fold larger areas than mouse astrocytes and displayed more complex structures (Fig. 3L-m, Fig. S3j). Thus, transplanted hiPSC-astrocytes were able to keep their intrinsic properties and develop in a cell-autonomous way adopting human-specific features and morphologies within the mouse host brain.

**Table 2:** Details of Human Cases.

Case number	Age	Gender	Aβ phase	Braak stage	PMI	Neuropathological Diagnosis	Type of dementia
1	82	M	5	3	72	AD	AD
2	81	F	5	5	48	AD, CAA, I	AD
3	85	M	5	3	48	AD, CAA, MI	AD-VaD
4	83	M	5	5	24	AD, CAA, I, B	AD-VaD
5	83	F	4	4	24	p-preAD, AGD, CM	0
6	85	F	4	3	24	p-preAD	0
7	87	M	4	3	96	p-preAD, CAA	0
8	72	M	2	3	72	p-preAD, I	0
9	75	M	1	2	48	p-pre-AD, AGD	0
10	66	F	0	0	48	non-AD control, AGD	0
11	62	M	0	0	48	non-AD control	0
12	64	M	0	0	24	non-AD control	0

The table shows the human subjects studied for histology of astrocytes. Indicated are: the age in years, the gender, the Aβ-MTL phase representing the distribution of Aβ deposits in the subfields of the MTL [43], the stage of neurofibrillary tangle pathology according to Braak and Braak [44] (NFT stage), PMI, neuropathological diagnosis and type of dementia. F female, M male, AD Alzheimer’s disease, AD-VaD Alzheimer’s disease plus signs of vascular dementia, p-preAD preclinical AD, non-AD non-demented control, AGD argyrophilic grain disease, B bleeding, CAA cerebrovascular angiopathy, CM carcinoma metastasis, I infarction, MI microinfraction, PMI post-mortem interval

### Human Astrocytes Undergo Morphological Changes Becoming Hypertrophic or Atrophic in Response to Amyloid-β Plaques

**Fig. 5:**
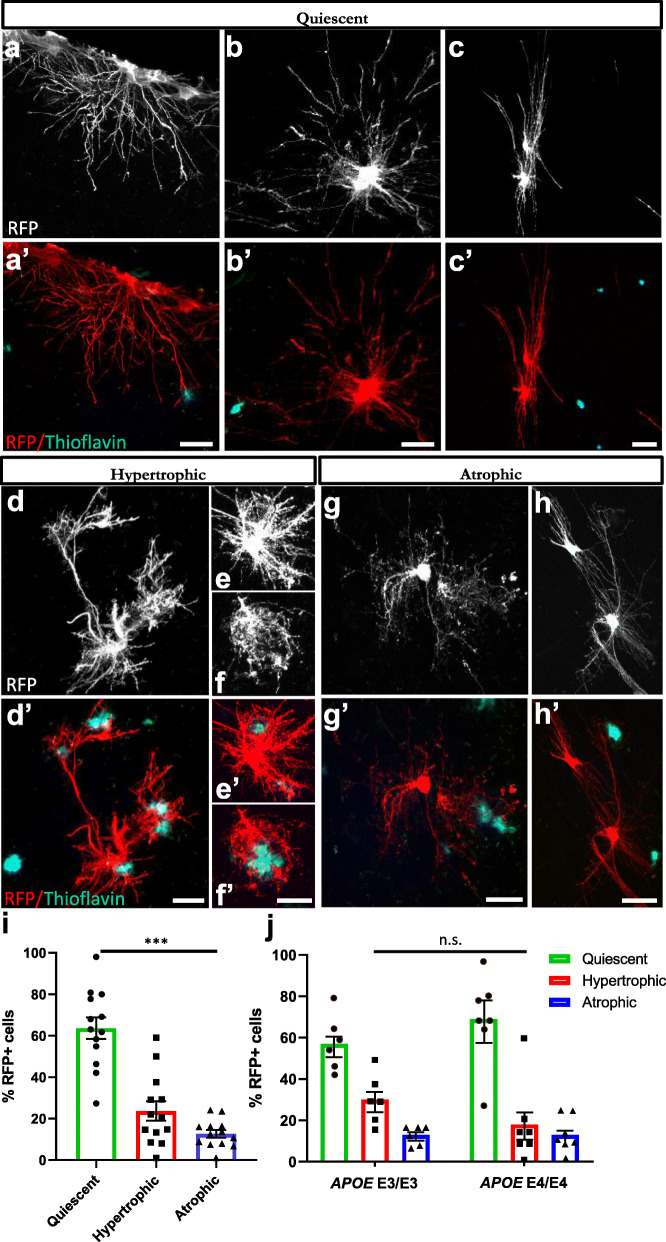
Human astrocytes go through morphological transformations becoming hypertrophic or atrophic in response to Aβ plaques within the chimeric AD brain.** (a-h, a’-h’)** hiPSC-astrocytes (RFP+, white or red) exposed to Aβ plaques (Thioflavin, green) show quiescent (**a-c, a’-c’**), hypertrophic (**d-f, d’-f’**) and atrophic (**g-h, g’-h’**) morphologies in chimeric AD mice five months after transplantation. Scale bars: 25 μm. **(i)** Percentage of hiPSC-astrocytes remaining quiescent, or becoming hypertrophic or atrophic as a group (n = 13 mice). Data are represented as mean ± SEM, one-way ANOVA with Friedman test, ***p < 0.001. **(j)** Percentage of hiPSC-astrocytes remaining quiescent, or becoming hypertrophic or atrophic per *APOE* genotype (n = 6 mice for *APOE* E3/E3; n = 7 mice for *APOE* E4/E4) five months post-transplantation. Data are represented as mean ± SEM, Chi-square test: n.s., non-significant

**Fig. 6:**
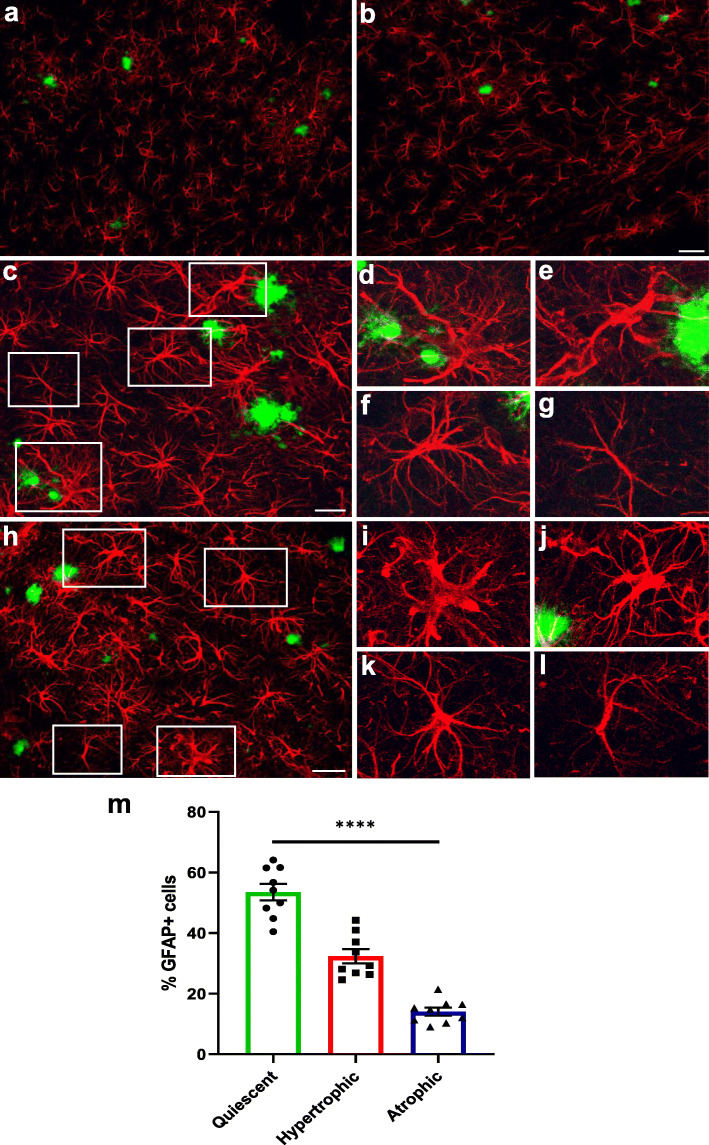
Endogenous mouse astrocytes become hypertrophic or atrophic in the chimeric AD brain.** (a-l)** Representative immunofluorescence images of GFAP+ mouse astrocytes (red) around amyloid-deposits (Thioflavin, green) in the cortex of chimeric AD brains. **(c-l)** GFAP+ mouse astrocytes (red) show hypertrophic (**d**-**e**, **i**-**j**), quiescent (**f**, **k**) and atrophic (**g**, **l**) morphologies close to amyloid deposits. (**d**-**g**, **i**-**l**) Enlarged views of the insets in **c** and **h**, respectively. Scale bars: 50 μm in (**a**, **b**) and 25 μm in (**c**, **h**). **(m)** Percentage of GFAP+ mouse astrocytes showing quiescent, hypertrophic or atrophic morphologies in the cortex of chimeric AD brains (*n* = 9 mice). Data are represented as mean ± SEM, one-way ANOVA with Friedman test, ****p < 0.0001

**Fig. 7:**
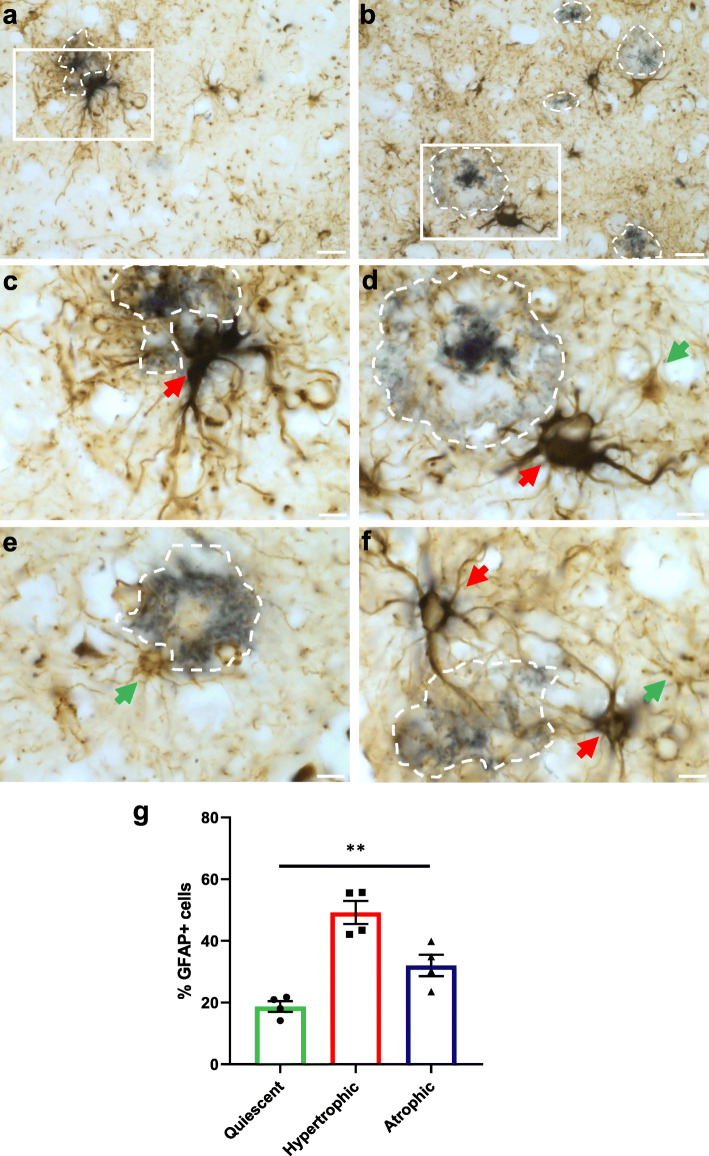
Human astrocytes in the brain of AD patients undergo morphological changes in response to Aβ.** (a-f)** Representative immunohistochemistry images of GFAP+ astrocytes (brown) around amyloid-deposits (blue, dashed lines) in the cortex and hippocampus of AD patient brains. **(a-d)** Overviews (**a**, **b**) and enlarged views (**c**, **d**) of the insets in **a**, **b**, respectively. **(c-f)** GFAP+ hypertrophic (red arrows) and quiescent or atrophic (green arrows) astrocytes around amyloid-deposits. Scale bars: 25 μm in (**a**, **b**); 10 μm in (**c**-**f**). **(g)** Percentage of GFAP+ human astrocytes displaying quiescent, hypertrophic or atrophic morphologies in the entorhinal cortex and hippocampus of AD patient brains (*n* = 4 AD patients). Data are represented as mean ± SEM, one-way ANOVA with Friedman test, **p < 0.01

We analyzed whether transplanted hiPSC-astrocytes responded to the presence of amyloid plaques in the brains of chimeric AD mice five months after transplantation, when the Aβ load is high. Immunofluorescence analyses with RFP revealed that 64 % of the astrocytes showed same morphologies (interlaminar, protoplasmic, varicose and fibrous) that we observed in the chimeric WT brains. We grouped these astrocytes under the term “quiescent” as they did not seem morphologically affected by the presence of Aβ plaques (Fig. 5a-c and a’-c’, Fig. 5i). Interestingly, we detected a subset of approximately 24 % of human astrocytes showing hypertrophic morphologies and thicker processes surrounding Aβ deposits (Fig. 5d-f and d’-f’, Fig. 5i). We also found another subset of ~ 12 % of human astrocytes showing atrophic features, displaying thinner processes that in some cases even looked degenerating (Fig. 5g-h and 5g’-h’, Fig. 5i). Human hypertrophic or atrophic astrocytes were phenotypes exclusively found in AD mice and not present in WT mice. These morphological alterations did not differ in *APOE* E3/E3 compared to *APOE* E4/E4 astrocytes five months after transplantation (Fig. 5j). Endogenous mouse astrocytes (RFP-) displayed similar alterations in the cortex of chimeric AD mice (Fig. 6a-l), with 54 % astrocytes showing quiescent, 32 % hypertrophic, and 14 % atrophic morphologies (Fig. 6m). Hypertrophic, atrophic and quiescent astrocytes were also found in close proximity to Aβ deposits in the entorhinal cortex and hippocampus of AD patients (Fig. 7a-f and Fig. S4), being hypertrophic astrocytes the most abundant population (49 % of the GFAP+ cells), followed by atrophic astrocytes (32 %) and quiescent ones (19 %) (Fig. 7g).

In conclusion, a proportion of the engrafted human astrocytes underwent morphological transformations becoming hypertrophic or atrophic in response to Aβ plaques. Similar alterations are observed in the human AD patients’ brains with higher proportions of hypertrophic and atrophic astrocytes, probably indicating that human astrocytes in the chimeric AD brains were at an earlier disease stage. The potential of astrocytes to become hyper- or a-trophic, or remain in a quiescent state, did not seem to be influenced by the *APOE* genetic background at this stage.

## Discussion

Interspecies differences pose a major challenge to model AD, highlighting the need of human-based models for elucidating pathogenic mechanisms. Studies using transplanted human microglia [32, 33] or neurons [31] in suitable AD model mice have made clear that human cellular context is critical. Here we continued these efforts and generated chimeric AD mice with transplanted human astrocytes.

We investigated in the current study the potential of such experiments using patient derived iPSC lines and isogenic counterparts (Table 1). We demonstrated integration of human astrocyte progenitors into the mouse brain and differentiation of the majority of cells into four main subtypes of human astrocytes expressing main astrocytic markers and showing human-specific large, complex morphologies and electrophysiological properties. Additionally, hiPSC-astrocytes were able to contact blood vessels and couple via gap-junctions with mouse cells, demonstrating functional integration in the host brain. In contrast to other glia chimeric models [35], we did not see replacement of the endogenous murine counterparts.

hiPSC-astrocytes responded to Aβ pathology showing morphological modifications and becoming hypertrophic or atrophic in the chimeric AD brain. Endogenous mouse astrocytes and human astrocytes in AD patients’ brains also underwent these morphological changes, as previously observed [9–11, 45]. The proportion of human astrocytes undergoing morphological transformations and becoming hyper- or a-trophic was lower in the chimeric AD brains than in the brains of AD patients, indicating that transplanted human astrocytes were most probably capturing the morphological phenotypes of astrocytes at an early stage of the disease and thus providing an optimal model to further study astrocytic changes at early stages of AD pathology. While astrocyte hypertrophy represents in principle a defensive response aimed at counteracting pathology, astrocyte atrophy might underlie loss of function with reduced homeostatic support and deficient neuroprotection [46]. Ageing itself is associated with an increase in astrocyte atrophy [47], which might favor the onset and/or evolution of AD and other age-dependent neurodegenerative diseases.

Such hypertrophic and atrophic responses did not seem to depend on the *APOE* genetic background in AD chimeras 5 months after transplantation. While these results also suggest that transplanted hiPSC-astrocytes are most probably at an early disease state, further work is needed to evaluate whether more subtle *APOE*-related molecular or functional alterations in astrocytes occur.

In the future it will be interesting to perform RNA sequencing analyses at single-cell resolution to dissect the cellular states of transplanted astrocytes at this stage of disease. At this moment, due to variations in engraftment efficiency (Fig. S1d) combined with the difficulty of recovering the engrafted cells from the mouse brain, transcriptomic analyses were not possible and further optimization of the protocol is needed.

Previously we and others also observed variations in transplantation efficiencies of hiPSC-derived microglia and neurons [32, 48, 49] which improved upon further experimentation [50]. Regarding astrocytes specifically, many reports have claimed the generation of successful “glia” chimeric mice [23, 34–36, 51]. These glia chimeras developed, in addition to human astrocytes, a large number of human NG2 cells and oligodendrocytes, whose relative ratios varied considerably across different brain regions and animals [23, 34, 51]. This suggests that in these other experiments a different state of glia precursors was transplanted which maintained more ‘stem cell like’ properties allowing these cells to spread over the brain and to compete with mouse glia as shown before [51]. We speculate that in our experimental conditions we transplanted astrocyte progenitors which were closer to a final astrocyte phenotype and therefore not able to proliferate once they were injected. It will now be critical to define the optimal window for transplantation of differentiating hiPSC-derived astrocyte progenitors in order to maximize astrocyte colonization of the mouse brain. In other experiments we succeeded already to determine this for microglia using the Migrate protocol [50]. In the Migrate protocol, there is a very critical window during the cell differentiation *in vitro* that results in 60–80 % chimerism. One week longer in culture results in < 5 % chimerism although the cells before transplantation look morphologically identical to the more efficiently transplanted ones. Other possible improvements would be the use of RAG2-/- mice. While this is likely not going to improve chimerism, these mice can be maintained for a much longer time than the NOD-SCID mice we used here.

## Conclusions

In conclusion, despite some intrinsic limitations, the approach to transplant human astrocytes into the mouse brain to study astrocyte pathophysiology in AD is promising. We recapitulated here typical morphological subtypes of human astrocytes that retained human-specific morphological and physiological properties and integrated functionally within the mouse host brain. In Alzheimer´s chimeric brains, transplanted astrocytes underwent morphological changes, showing hypertrophic and atrophic phenotypes in response to amyloid plaques. These first analyses establish the basis for further molecular and functional studies of human astrocytes in an *in vivo* context. Moreover, the combination of the model with isogenic *APOE* lines points out the potential use of this approach to analyze the impact of patient-derived and genetically modified astrocytes on human CNS diseases.

## Methods

### Generation of isogenic CRISPR/Cas9 gene-edited hiPSCs

Eight hiPSC lines were generated previously from three *APOE* E4/E4 carriers diagnosed with AD (Table 1) [52]. The correct *APOE* sgRNA sequence orientation was confirmed by Sanger sequencing and CRISPR/Cas9-*APOE* sgRNA plasmid cleavage efficiency was determined using the Surveyor mutation detection kit in 293T cells. The single-strand oligo-deoxynucleotide (ssODN) was designed to convert *APOE* E4/E4 to *APOE* E3/E3 with a protospacer adjacent motif (PAM) silent mutation to prevent recurrent Cas9 editing. hiPSCs (70–80 % confluent) dissociated by Accutase supplemented with 10 µM Thiazovivin (Tzv) (Millipore), were harvested (200 x g, 3 min), and electroporated (Neon®, ThermoFisher) according to the manufacturer’s instructions. In brief, cells resuspended in 10 µl Neon Resuspension Buffer R, 1 µg CRISPR/Cas9-*APOE* sgRNA plasmid and 1 µl of 10µM of ssODN were electroporated plated on Matrigel-coated plates in mTeSR media with 10 µM Tzv for 72 h. GFP-expressing hiPSC were isolated by FACS (BD FACSAria). Sorted single cells were suspended in mTeSR with Tzv and plated into 96 well plates containing MEFs (4,000 cells/well). Clones were expanded and transferred to a replicate plate for gDNA isolation and Sanger sequencing to identify genome edited clones.

### Karyotyping

G-banding karyotyping was performed by Wicell Cytogenetics (Madison, WI). Karyotypes are shown in Fig. S1a.

### Generation of reporter hiPSC-astrocytes

The consent for reprogramming human somatic cells to hiPSC was carried out on ESCRO protocol 19 − 04 at Mount Sinai (J.TCW.). hiPSCs maintained on Matrigel (Corning) in mTeSR1 (StemCell Technologies) supplemented with 10 ng/ml FGF2 StemBeads (StemCultures) were differentiated to neural progenitor cells (NPCs) by dual SMAD inhibition (0.1µM LDN193189 and 10µM SB431542) in embryoid bodies (EB) media (DMEM/F12 (Invitrogen, 10,565), 1x N2 (Invitrogen, 17502-048), and 1x B27-RA (Invitrogen, 12587-010)). Rosettes were selected at 14 DIV by Rosette Selection Reagent (StemCell Technologies) and patterned to forebrain NPCs with EB media containing 20ng/ml FGF2 (Invitrogen). NPCs (CD271^−^/CD133^+^) were enriched by magnetic activated cell sorting (Miltenyi Biotec) [53] and validated immunocytochemically using SOX2, PAX6, FoxP2 and Nestin (Table S1). Dissociated single cell forebrain NPCs were plated 1,000,000 cells/well on 12 well plates and transfected with lentiGuide-tdTomato (Addgene #99,376) plasmid and selected by hygromycin. Pure fluorescent expressing NPCs were plated at low density (15,000 cells/cm^2^) on matrigel coated plates and differentiated to astrocytes in astrocyte medium (ScienCell, 1801) as described [38]. Cells were cultured and harvested as astrocyte progenitors at DIV 40–44, validated immunocytochemically and/or by FACS for the astrocyte-specific markers and used for subsequent experiments.

### AD and WT Immunodeficient Mice

Mice were generated as described previously [31]. Briefly, *APP PS1* tg/wt mice (expressing KM670/671NL mutated *APP* and L166P mutated *PS1* under the control of the Thy1.2 promoter1.1) [39] were crossed with the immunodeficient NOD-SCID mice (NOD.CB17-*Prkdc*^scid^) that carry a single point mutation in the *Prkdc* gene [40]. *APP PS1* tg/wt *Prkdc*^scid/+^ mice from the F1 generation were crossed with NOD-SCID mice to generate *APP* *PS1* tg/wt *Prkdc*^scid/scid^ immunodeficient mice. *APP PS1* tg/wt *Prkdc*^scid/scid^ mice were subsequently crossed with NOD-SCID mice to generate either *APP PS1* tg/wt *Prkdc*^scid/scid^ (AD mice) or *APP PS1* wt/wt *Prkdc*^scid/scid^ (WT mice) used for transplantations. Mice were housed in IVC cages in a SPF facility; light/dark cycle and temperature were always monitored. After weaning, no more than five animals of the same gender were kept per cage. Genotyping was done as previously described [31]. Transplantation experiments were performed in both male and female littermates at P0-P4. Mouse work was performed in accordance with institutional and national guidelines and regulations, and following approval of the Ethical Committee of the KUL. All experiments conform to the relevant regulatory standards.

### Intracerebral Grafting

Grafting experiments of hiPSC-derived astrocyte progenitors using neonatal *APP PS1* tg/wt NOD-SCID (AD mice) and *APP PS1* wt/wt NOD-SCID (WT mice) at postnatal days P0-P4 were performed as described previously [31] with some modifications. Briefly, hiPSC-derived astrocyte progenitor cells at DIV 44 were enzymatically dissociated, supplemented with HB-EGF (100 − 47, Peprotech) and RevitaCell (A2644501, ThermoFisher) and injected into the frontal cortex of AD or WT mice. The pups were anesthetized by hypothermia and about 200,000 cells were injected with Hamilton syringes into the forebrain at two locations: 1 mm posterior Bregma, 1.5 mm bilaterally from the midline and 1.2 mm from the pial surface. Transplanted pups were returned to their home cages until weaning age.

### Electrophysiological Characterization of Human Glia in Chimeric Mice

Four to five month-old WT mice were anesthetized with isoflurane and decapitated. Acute 300 μm-thick coronal slices were cut on a Leica VT1200 vibratome in a sucrose-based cutting solution consisting of (mM): 87 NaCl, 2.5 KCl, 1.25 NaH2PO4, 10 glucose, 25 NaHCO3, 0.5 CaCl2, 7 MgCl2, 75 sucrose, 1 kynurenic acid, 5 ascorbic acid, 3 pyruvic acid (pH 7.4 with 5 % CO2/ 95 % O2). Slices were allowed to recover at 34ºC for 45 min and maintained at room temperature (RT) in the same solution for at least 30 min before use. During recordings, slices were submerged in a chamber (Warner Instruments) perfused with 3-4 mL/min artificial cerebrospinal fluid (ACSF) consisting of (mM): 119 NaCl, 2.5 KCl, 1 NaH2PO4, 26 NaHCO3, 4 MgCl2, 4 M CaCl2, 11 glucose at pH 7.4 with 5 % CO2/ 95 % O2. Recordings were done at 34ºC. hiPSC-astrocytes were identified based on the td-Tomato fluorescence with a 40x objective in an epifluorescent microscope (Zeiss Axio Examiner.A1). Whole-cell current clamp recordings were made from 17 hiPSC-astrocytes (hiPSC lines #1 to #4, n = 6 mice) with borosilicate glass recording pipettes (resistance 3-6MΩ). Pipettes were pulled on a horizontal micropipette puller (Sutter P-1000) and filled with a K-gluconate based internal medium consisting of (mM): 135 K-Gluconate, 4 KCl, 2 NaCl, 10 HEPES, 4 EGTA, 4 MgATP, 0.3 NaATP (pH 7.25). To post-hoc identify the patched astrocyte and analyze its potential to form gap-junctions, 40 µM Alexa Fluor hydrazide dye 488 (Invitrogen) was included in the internal medium. Current steps of incrementing 20 pA were injected starting from 50 pA up to 150 pA. Resting membrane potential was calculated using Clampfit 10.7 (Axon Instruments). Currents were sampled at 20 kHz and stored after 3 kHz low-pass Bessel filtering. The data was low-pass filtered at 1 kHz (Molecular devices DigiData 1440 A and Multiclamp 700B). Pipette series resistance and membrane holding current were monitored throughout all recordings to ensure stability of the recording.

### Immunofluorescence (IF) in Chimeric Mice

For IF analysis, mice were euthanized with CO_2_ and perfused with phosphate-buffered saline followed by 4 % paraformaldehyde solution. The brain was then removed, post-fixed in the same fixative overnight to 48 h and cut into 40 μm slices on a Leica VT1000S vibratome. IF on grafted brains was performed as described previously [31] using primary and secondary antibodies (Table S1). Antigen retrieval was performed by microwave boiling the slides in 10mM tri-Sodium Citrate buffer pH 6.0 (VWR). Aβ plaques were detected by staining with Thioflavin (Sigma). Briefly, for Thioflavin staining brain sections were incubated with a filtered 0.05 % aqueous Thioflavin-S (Sigma) solution in 50 % ethanol for 5 min at RT and rinsed gradually with 70 %, 95 % ethanol and water. Nuclei staining was performed using a specific anti-human Nuclear Antigen antibody (hNuclei) (Table S1), the pan-nuclear staining TOPRO3 (Invitrogen), or DAPI (Sigma). The sections were mounted with Glycergel (DAKO). Confocal images were obtained using a Nikon Ti-E inverted microscope equipped with an A1R confocal unit driven by NIS (4.30) software. The confocal was outfitted with 20 × (0.75 NA), 40x oil (1.4 NA) and 60x oil (1.4 NA) objectives lenses. For excitation 405 nm, 488 nm, 561 nm, 638 nm laser lines were used.

### Neuropathology on Human Brain Samples

Brain tissue samples from 4 AD, 5 pre-AD and 3 non-demented control patients were included in this study (Table 2). The autopsies were performed with informed consent in accordance with the applicable laws in Belgium (UZ Leuven) and Germany (Ulm, Bonn and Offenbach). The use of human tissue samples for this study was approved by the UZ Leuven ethical committee (Leuven, Belgium). Brain tissues were collected as described in previous studies [54] with an average post-mortem interval (PMI) of 48 h. Briefly, after autopsy, the brains were fixed in 4 % aqueous solution of formaldehyde for 2–4 weeks. Samples of the anterior entorhinal cortex and hippocampus were dissected coronally, dehydrated and embedded in paraffin. The paraffin blocks were microtomed at 10 μm, mounted on Flex IHC adhesive microscope slides (Dako), and dried at 55 °C before storing. For neuropathological analysis, sections from all blocks were stained with anti-pTau (AT8), anti-Aβ (4G8) (Table S1), and with the Gallyas and the Campbell-Switzer silver techniques for detection of neurofibrillary changes and amyloid deposits [43].

The post-mortem diagnosis of AD pathology was based upon the standardized clinico-pathological criteria, including the topographical distribution of Aβ plaques in the medial temporal lobe (AβMTL phase) based on Aβ immunohistochemistry [43], and the Braak neurofibrillary tangle (NFT) stage based on pTau immunohistochemistry [44]. The study comprised 12 cases with an average age of 77 years and a female to male ratio of 4:8. The cases were divided in three groups based on the clinical and neuropathological diagnosis: (1) AD = high-intermediate degree of AD pathology and signs of cognitive decline during life (CDR ≥ 0.5); (2) p-preAD = cases with intermediate-low degrees of AD pathology lacking clinical signs of cognitive decline (CDR = 0); (3) non-AD = low-no pathological signs of AD pathology (CDR = 0).

### Immunohistochemistry and Immunofluorescence on Human Samples

The distribution of astrocytes and Aβ deposits was examined in human samples of the entorhinal cortex and hippocampus using immunohistochemical and immunofluorescence techniques. Immunohistochemical detection of Aβ deposits and astrocytes was performed after formic acid pretreatment. For double-labeling, a monoclonal anti-Aβ_17−24_ antibody (4G8, Table S1) was subsequently combined with a polyclonal anti-GFAP (DAKO, Table S1) as described previously [54]. The anti-Aβ_17−24_ antibody was detected with biotinylated secondary antibodies and ABC, and visualized with 3,3´diaminobenzidine-HCl. After peroxidase blocking, the anti-GFAP was applied, detected with biotinylated secondary antibodies, and ABC, and visualized with the Vector peroxidase kit SG (blue staining). Microscopy analysis was performed using a light Leica DM2000 LED microscope (Leica Microsystems) and images were captured with a Leica DFC7000 T camera (Leica Microsystems).

For double-labeling immunofluorescence, sections were pre-treated as mentioned above and incubated with formic acid for 3 min, when required. Immunostainings were performed with an antibody cocktail and primary antibodies were detected with species-specific fluorescent-conjugated secondary antibodies (Table S1). Images were captured via Nikon NIS-Elements software using a Nikon A1R laser scanning confocal system coupled to a Nikon Eclipse Ti inverted microscope (Nikon Instruments, Inc.). Acquired data were further processed using ImageJ software (National Institutes of Health).

### Quantification and Statistical Analysis

Morphometry and measurements were performed with Fiji/ImageJ software on animals at five months after transplantation. At least 4–5 different coronal brain sections comprising the transplanted astrocytes and the mouse host tissue were included per animal. Immunofluorescence (IF) sections were imaged by confocal microscopy (Nikon Ti-E inverted microscope) using a 20 × (0.75 NA) objective lens to image Z-stacks (8–10 optical sections with a spacing of 1 μm). All images were acquired using identical acquisition parameters as 16-bit, 1024 × 1024 arrays. Maximum intensity projections and threshold were applied using Fiji/ImageJ to isolate specific fluorescence signals.

For analyses of cell integration, brains were sectioned and stained with the antibodies against RFP and hNuclei (human Nuclear antigen). The number of hNuclei+ and RFP+ cells was counted manually on IF images of astrocytes derived from the eight hiPSC lines used on the study (#1 to #8, Table 1) in WT and AD mice. Final counts were corrected for series number (1:6) to get an estimate of the total number of hNuclei+ and RFP+ cells per animal (Fig. S1).

For analyses of cell identity, brains were sectioned and stained with the following antibodies: RFP and hNuclei (human Nuclear antigen), GFAP (astrocyte marker), NeuN (neuronal marker) or APC (marker of oligodendrocytes). Results are shown for four hiPSC lines (#1, #2, #7 and #8, Table 1) in WT and AD mice. Total percentages of RFP+ cells co-localizing with GFAP (n = 14 mice), hNuclei (n = 15 mice), NeuN or APC (n = 9 mice each) were manually determined on IF images using Fiji/ImageJ. Data are represented as mean ± SEM. Statistical analyses were done with Student’s t test (Fig. 1 and Fig. S3).

To analyze the morphological subtypes of hiPSC-astrocytes in WT mice, brains were sectioned and stained with antibodies against RFP and hNuclei (human Nuclear antigen) and morphometry analyses were manually performed on IF images using Fiji/ImageJ. Results are shown for four hiPSC lines (#1, #2, #7 and #8, Table 1) in WT mice (n = 14). Data are represented as mean ± SEM (Fig. 3).

For quantification of the average cell area, brains were stained with RFP and GFAP, and the NIS-elements software was used (version 5.21.01 build 1483, Nikon Instruments). All the z-stacks were first denoised (denoise.ai tool) and then projected on a 2D image using an extended focus operation (EDF, zero-based, balanced). The resulting 2D image was used for further quantification with a General Analysis (GA3) protocol. In short, to count the number of cells, a spot detection approach was used (average size 11 μm). For detection of the cell area, we first applied a rolling ball filter (6 μm) and, consequently, a thresholding step. Both the settings for the threshold and the spot detection were adjusted per image to compensate for differences in intensity due to a change of acquisition parameters. Results are shown for four hiPSC lines (#1, #2, #3 and #4, Table 1) in WT and AD mice (n = 6 WT and n = 6 AD mice). Data are represented as mean ± SEM. Statistical analysis was done with Student’s t test (Fig. 3).

To analyze the morphological responses of hiPSC-derived and endogenous mouse astrocytes to Aβ plaques in chimeric AD mice, brains were sectioned and stained with RFP, GFAP and Thioflavin, and morphometry analyses were manually performed on IF images using Fiji/ImageJ. Results are shown for four hiPSC lines (#1, #2, #7 and #8, Table 1) in AD mice (n = 13; Fig. 5); and for endogenous astrocytes in AD mice (*n* = 9; Fig. 6). Data are represented as mean ± SEM. Statistical analysis was performed with one-way ANOVA with Friedman test (Figs. 5 and 6) and Chi-square test (Fig. 5).

To analyze the morphological phenotypes of human astrocytes in the brain of AD patients, paraffin sections from the anterior entorhinal cortex and hippocampus were stained with an anti-Aβ_17−24_ antibody and GFAP and morphometry analyses were manually performed on images using Fiji/ImageJ. Results are shown for four AD patients (subjects 1–4, Table 2) and data are represented as mean ± SEM (Fig. 7). Statistical analysis was performed with one-way ANOVA with Friedman test.

## Supplementary Information

**Additional file 1:**

## Data Availability

The datasets used and/or analyzed during the current study are available from the corresponding authors on reasonable request.

## Abbreviations

5 M: 5 months of age
AD: Alzheimer’s disease
Aβ: Amyloid-β
*APOE*: Apolipoprotein E
CDR: Clinical dementia rating
DIV: Days in vitro
EB: Embryoid bodies
hAPCs: Human astrocyte progenitor cells
hiPSCs: Human induced pluripotent stem cells
IF: Immunofluorescence
IVC: Individually ventilated cages
NFT: Neurofibrillary tangles
NPCs: Neural progenitor cells
PAM: Protospacer adjacent motif
PMI: Post-mortem interval
RFP: Red fluorescent protein
RT: Room temperature
SPF: specific pathogen free
ssODN: single-strand oligo-deoxynucleotide
Tzv: Thiazovivin
WT: Wild-type
