# Supplementary material for: Human iPSC-derived astrocytes transplanted into the mouse brain undergo morphological changes in response to amyloid-β plaques
Source: Mol Neurodegener. 2021 Sep 25;16:68. doi: 10.1186/s13024-021-00487-8 (PMC8467145; doi:10.1186/s13024-021-00487-8)

**Table S1. Antibodies used in this study.** The table summarizes information about supplier company, catalog number and concentration of use

| **Antibody** | **Company** | **Cat #** | **Concentration** |
| --- | --- | --- | --- |
| 4G8 | BioLegend | 800703 | 1/5000 |
| APC | Millipore | OP80 | 1/200 |
| AQP4  AT8 | Alomone Labs  Thermo Fisher Scientific | AQP-004  MN1020 | 1/300  1/100 |
| Cx43 | Santa Cruz | sc-271837 | 1/200 |
| EAAT1 (GLAST; ACSA-1)  FoxP2 | Miltenyi Biotec  Abcam | 130-095-814  Ab16046 | 1/200  1/100 |
| GFAP | Synaptic Systems | 173 004 | 1/1000 |
| GFAP | DAKO | Z033401-2 | 1/1000 |
| Glutamine Synthetase | Millipore | MAB302 | 1/100 |
| human GFAP | BioLegend | 837202 | 1/200 |
| human Nuclear Antigen  Nestin | Millipore  Abcam | MAB1281  Ab22035 | 1/100  1/200 |
| NeuN  PAX6 | Synaptic Systems  Abcam | 266 004  Ab5790 | 1/300  1/100 |
| RFP | Rockland | 600-401-379 | 1/1000 |
| RFP | Rockland | 200-301-379 | 1/1000 |
| S100b  SOX2 | Abcam  Cell Signaling | ab52642  3579S | 1/500  1/300 |
| STEM123 | Takara | Y40420 | 1/100 |
| Vimentin | BD Pharmingen | 550513 | 1/100 |
| Anti-Mouse 488 | Invitrogen | A21202 | 1/500 |
| Anti-Mouse 594 | Invitrogen | A21203 | 1/500 |
| Anti-Mouse 647 | Invitrogen | A31571 | 1/500 |
| Anti-Mouse biotinylated | Vector Laboratories | BA-9200 | 1/250 |
| Anti Mouse HRP | DAKO | P 0447 | 1/200 |
| Anti-Rabbit 488 | Invitrogen | A21206 | 1/500 |
| Anti-Rabbit 594 | Invitrogen | A21207 | 1/500 |
| Anti-Rabbit biotinlylated | Vector Laboratories | BA-1000 | 1/250 |
| Anti-Guinea Pig 647 | Jackson ImmunoResearch | 706-175-148 | 1/500 |

**Figure S1 Characterization of hiPSC lines, derived glial progenitors and engraftment capacity. (a)** Karyotype analysis of the hiPSC lines used for transplantation showing no genomic alterations. **(b)** hiPSC-derived glial progenitors at 44 days *in vitro* express main astroglial markers. Scale bar: 50 µm. **(c)** Coronal sections stained with RFP show the distribution of xenografted hiPSC-derived astrocytes on a chimeric mouse brain at five months post-transplantation. Scale bar: 200 µm. **(d)** Assessment of engraftment capacity of transplanted hiPSC-derived astrocytes: * indicates less than 5,000 RFP+ cells, ** between 5,000 and 25,000 RFP+ cells, and *** between 25,000 and 200,000 RFP+ cells in the chimeric mouse brain.


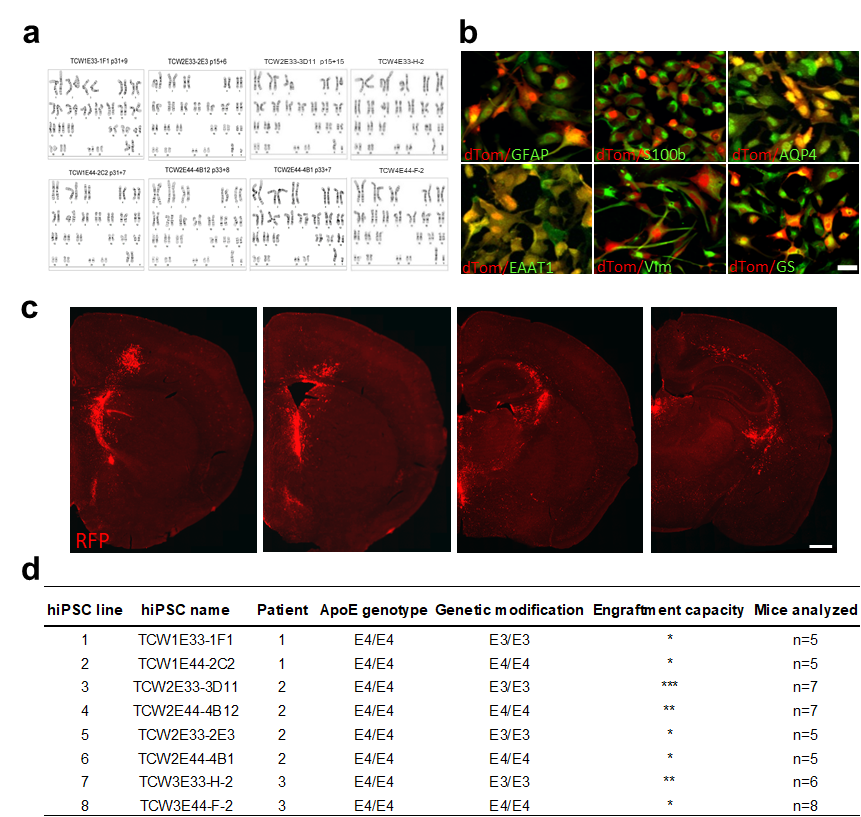


**Figure S2 Lentivirus gene delivery method does not activate inflammatory pathways. (a)** Images of hiPSC-derived astrocytes with control (no DNA), DNA transduction (lentivirus reporter, td-Tomato) and LPS treatment (50μg/ml) after 20 hrs. Scale bar: 100 µm. **(b)** Inflammatory cytokine (IL6) secretion from conditioned medium of astrocytes transduced with/without DNA and LPS treatment for 20hrs. **(c)** Relative levels of key inflammatory cytokine expression (IL6, CXCL2, IL1a, and IL1b) in each condition. Results suggest that there are no significant differences before and after gene delivery via lentivirus with multiplicity of infection (MOI) = 2. Data are represented as mean ± SEM. One-way ANOVA test with * p < 0.05, ** p < 0.01, *** p < 0.001, n.s. represents not significant.


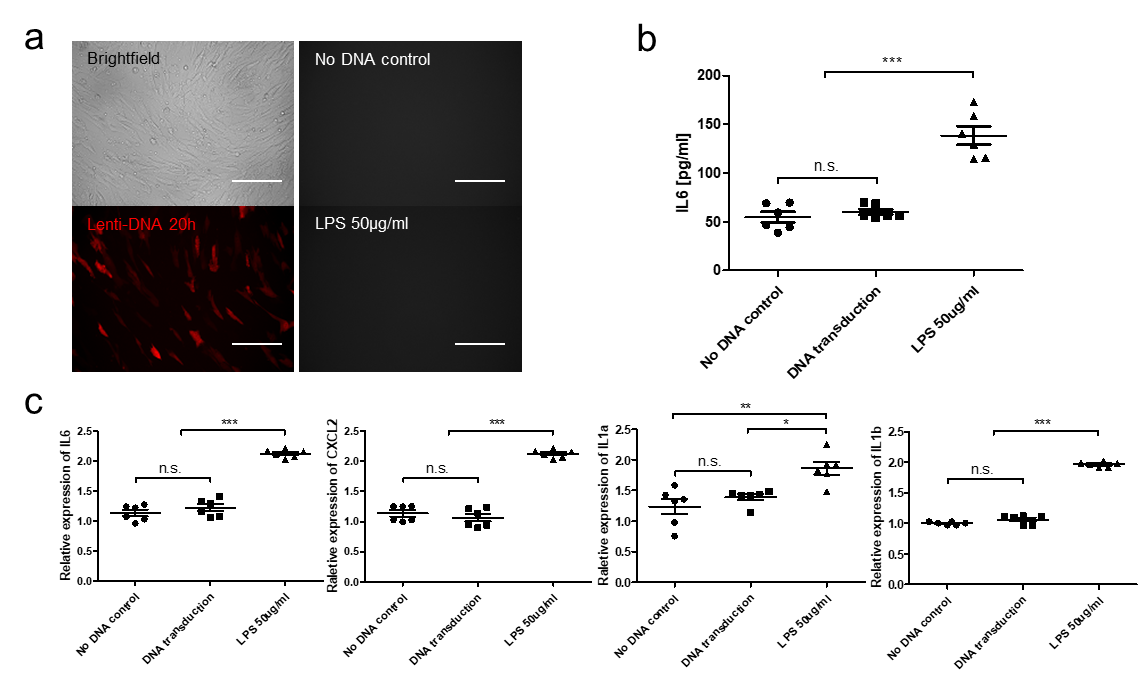


**Figure S3 Characterization of hiPSC derived glia *in vivo*. (a-c)** Five months after transplantation, hiPSC-glia (RFP+, red) express human GFAP (a; hGFAP, green) but not the neuronal marker NeuN (b; green) or the oligodendrocyte marker APC (c; green). Scale bars: 25 µm. **(d-f)** Percentage of RFP+ cells expressing GFAP (d; n=7 mice for *APOE E3/E3*; n=7 mice for *APOE E4/E4*; n=4 wild-type; n=4 AD mice), NeuN (e; n=6 mice for *APOE E3/E3*; n=3 mice for *APOE E4/E4*) or APC (f; n=6 mice for *APOE E3/E3*; n=3 mice for *APOE E4/E4*). Data are represented as mean ± SEM, Student’s t test: n.s., non-significant. **(g-i)** Overview (g) and representative images (h-i) of RFP+ and GFAP- progenitor cells showing morphological features of radial glia. Scale bars: 25 µm. **(j)** hiPSC-astrocytes (RFP+ GFAP+) and mouse astrocytes (RFP- GFAP+) coexist within chimeric brains. Scale bar: 25 µm.


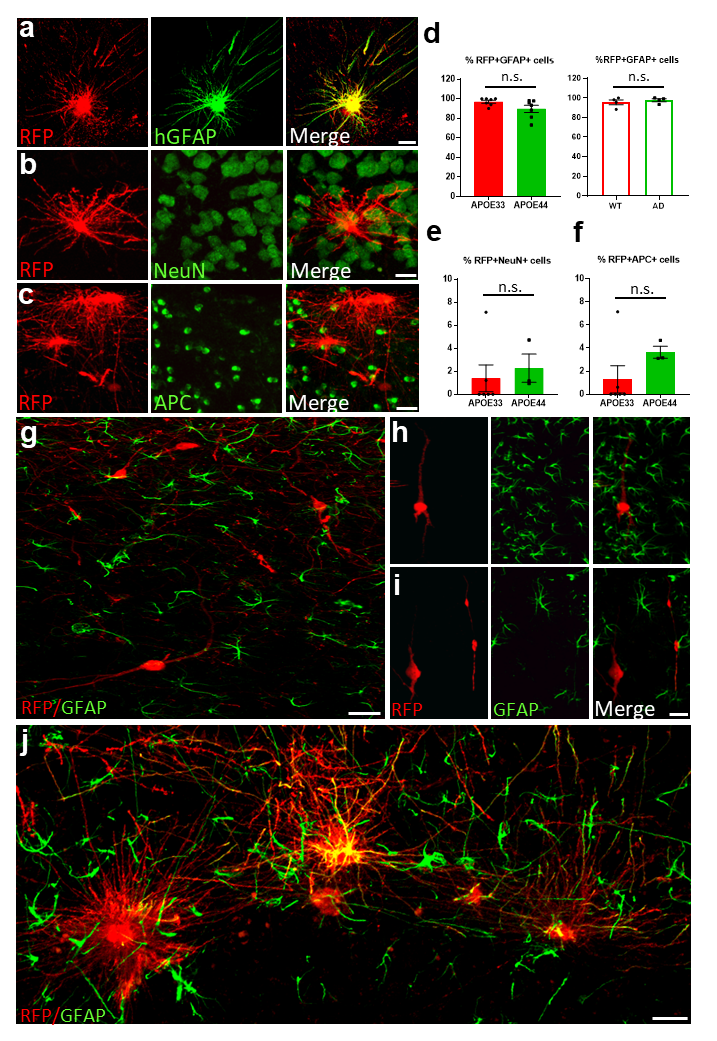


**Figure S4** **Hypertrophic, quiescent and atrophic astrocytes close to amyloid deposits in human AD patient brains. (a-k)** Representative immunofluorescence images of GFAP+ astrocytes (red) around amyloid-deposits (4G8, green) in the cortex and hippocampus of AD patient brains. **(b-k)** GFAP+ astrocytes (red) show hypertrophic (c-d, h-i), quiescent (e, j) and atrophic (f, k) morphologies close to amyloid deposits. (c-f, h-k) Enlarged views of the insets in b and g, respectively. Scale bars: 50 µm in (a) and 25 µm in (b, g)


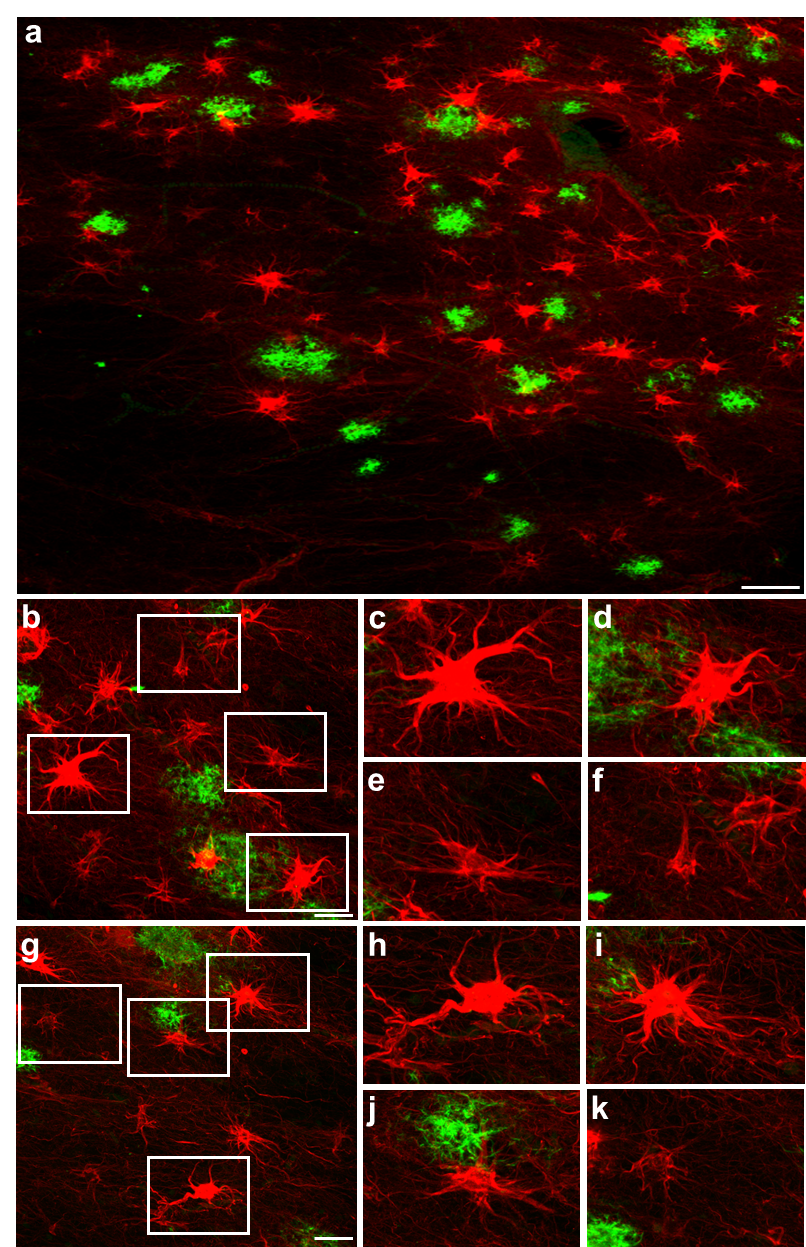

Supplement: Supplementary file 1 — Additional file 1: [file 13024_2021_487_MOESM1_ESM.docx]
